# Laparoscopy-Assisted Percutaneous Cholangiography in Biliary Atresia Diagnosis: Comparison with Open Technique

**DOI:** 10.1155/2016/5637072

**Published:** 2015-12-24

**Authors:** Murat Alkan, Kamuran Tutus, Ender Fakıoglu, Onder Ozden, Zehra Hatipoglu, Serdar Hilmi Iskit, Recep Tuncer, Unal Zorludemir

**Affiliations:** ^1^Department of Pediatric Surgery, Cukurova University Faculty of Medicine, Adana, Turkey; ^2^Department of Anesthesiology, Cukurova University Faculty of Medicine, Adana, Turkey

## Abstract

*Introduction*. Biliary atresia is a surgical cause of prolonged jaundice, which needs to be diagnosed with cholangiography that has traditionally been performed via laparotomy. Laparoscopic assistance has lately been introduced to avoid unnecessary laparotomy. We aim to evaluate the benefits of the laparoscopy-assisted cholangiography and compare it to the traditional procedure via laparotomy. *Patients and Method*. The medical records of the cases who had undergone cholangiography for prolonged jaundice between 2007 and 2014 were analyzed. The patients were grouped according to cholangiography technique (laparotomy/laparoscopy). The laparoscopy and laparotomy groups with patent bile ducts were focused and compared in terms of operation duration, postoperative initiation time of enteral feeding, and full enteral feeding achievement time. *Results*. Sixty-one infants with prolonged jaundice were evaluated between 2007 and 2014. Among the patients with patent bile ducts, operation duration, postoperative enteral feeding initiation time, and the time to achieve full enteral feeding were shorter in laparoscopy group. *Conclusion*. Laparoscopic cholangiography is safe and less time-consuming compared to laparotomy, with less postoperative burden. As early age of operation is a very important prognostic factor, laparoscopic evaluation should be an early option in work-up of the infants with prolonged jaundice with direct hyperbilirubinemia, for diagnosis/exclusion of biliary atresia.

## 1. Introduction

Prolonged jaundice is defined as jaundice longer than 14 days in term infants and 21 days in preterm infants. Over this period, prolonged jaundice with conjugated hyperbilirubinemia in a newborn with pale stool and dark urine alerts the pediatrician to investigate the cholestatic disorders such as infections due to congenital rubella, CMV, toxoplasmosis, and endocrine and metabolic disorders as hypothyroidism, alpha-1-antitrypsin deficiency, and aminoaciduria. After all these investigations, ultrasonography, hepatic scintigraphy, and usually liver biopsy take place. In cases of no diagnosis despite these investigations, surgical exploration is usually needed to exclude biliary atresia. Cholangiography via laparotomy or laparoscopy has remained as the gold standard for the diagnosis of biliary atresia. Invasiveness and high morbidity of explorative procedure generally make pediatricians refer the patient to the pediatric surgeon after their investigations exclude all nonsurgical causes of jaundice. On the other hand, early identification of biliary atresia affects the success rate of the operation and improves the outcome [1].

Laparoscopy is a minimally invasive procedure that can exclude surgical causes of jaundice and may help avoid unnecessary laparotomy in neonates.

Herein, we present our technique, laparoscopy-assisted percutaneous cholangiography with liver biopsy with a single umbilical trocar, and compared the results of this technique with traditional open surgical explorative cholangiography and liver biopsy.

## 2. Patients and Method

We performed a retrospective chart review of all infants who were referred between December 2007 and December 2014, with prolonged jaundice in whom clinical, radiological, and laboratory tests failed to get a definitive diagnosis and surgical evaluation was needed to exclude biliary atresia. Infants who had undergone Kasai portoenterostomy were excluded from the study. The patients with patent biliary duct systems were included in the study and these patients were divided into two groups: Group I, patients who had single-port laparoscopy-assisted percutaneous cholangiography and liver biopsy, and Group II, patients who had cholangiography and liver biopsy via laparotomy. Those groups were compared in terms of operation duration, postoperative enteral feeding initiation time, time to achieve full enteral feeding, and postoperative complications. Evaluation of hospital stay length and cost effectiveness was not possible, because most of the patients had been hospitalized in pediatric clinics long before their referral to our department (Pediatric Surgery), some with additional serious anomalies, so that the hospital stay lengths and hospitalization costs were far away from being attributable to the cholangiography techniques.

### 2.1. Statistical Methods

Normality was checked by Shapiro-Wilks test continuous variables. Nonparametric tests were chosen since the data were not distributed normally. Data were analyzed by Mann-Whitney *U* test and Chi Square test was used to evaluate the differences between groups. Data were expressed as *n* (%), mean ± SD, and median (min-max). A *p* value < 0.05 is considered significant. Statistical analysis was performed using the statistical package SPSS v 20.0.

### 2.2. Laparoscopic Procedure

**Figure 1:**
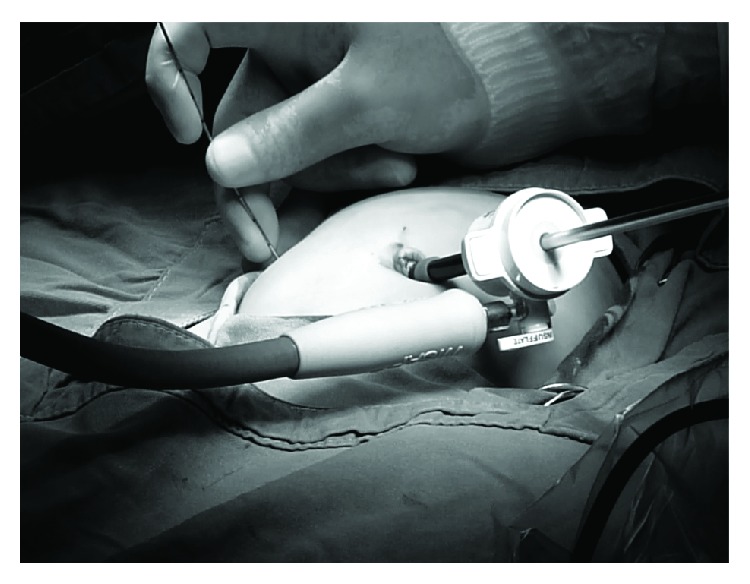
5 mm umbilical trocar is introduced and Chiba needle is inserted percutaneously.

**Figure 2:**
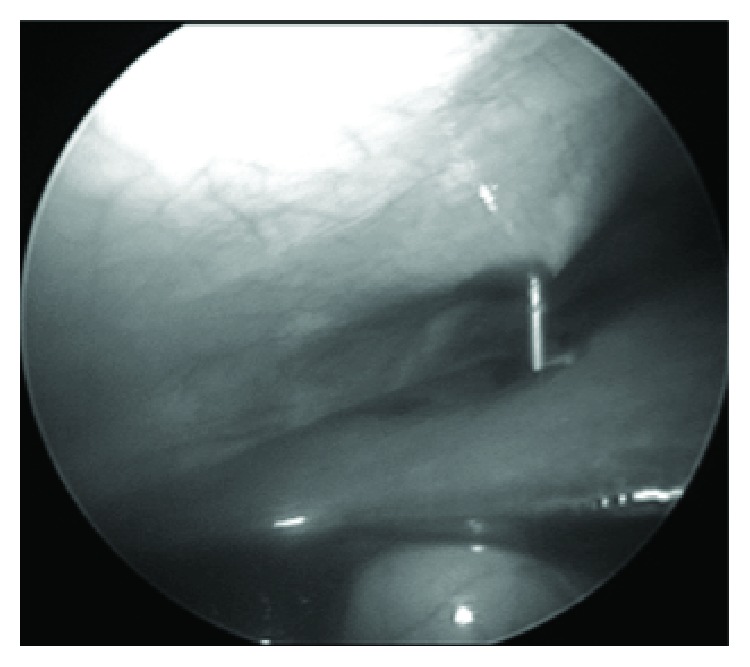
Chiba needle is inserted into the gallbladder via transpassing the liver under direct telescopic vision.

**Figure 3:**
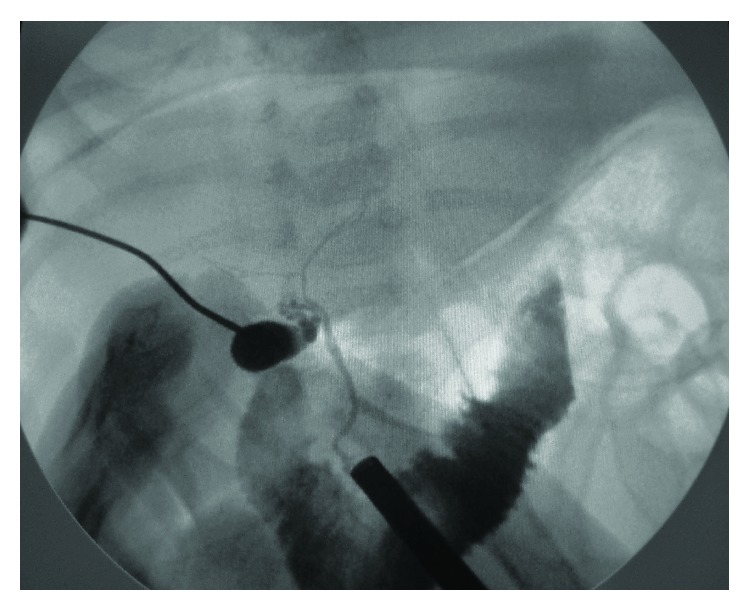
Cholangiography: passage of the contrast agent into the intrahepatic ducts and into the intestinal system excludes biliary atresia.

**Figure 4:**
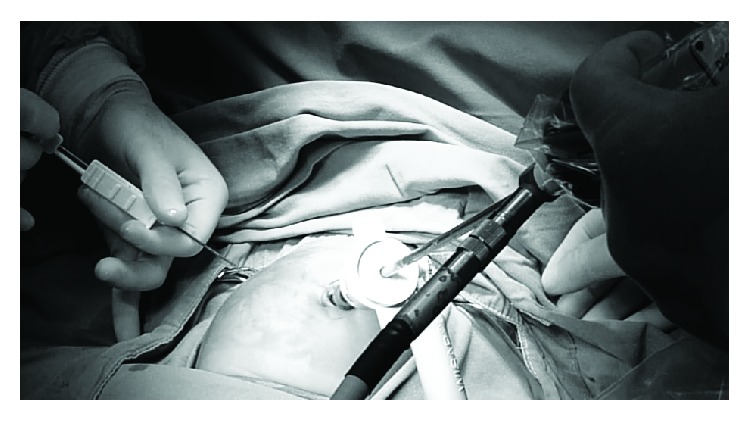
When the biliary ducts are patent, with the 18 G tru-cut biopsy needle, liver biopsy is taken under the vision of telescope.

Under general anesthesia, a 5 mm umbilical trocar was introduced via the open Hasson technique, in order to create a port for a 30-degree laparoscope. Carbon dioxide was insufflated to a pressure between 6 and 8 mmHg. The liver and gallbladder were examined under direct laparoscopic vision with the help of tilting the operating table 45-degree reverse-Trendelenburg. Under direct vision of the telescope, a Chiba needle was inserted percutaneously into the gallbladder via transpassing the liver without gallbladder dissection (Figures 1 and 2). A few milliliters of contrast agent was injected into the gallbladder while fluoroscopic radiograms are being obtained (Figure 3). If the biliary ducts were patent, with the 18G tru-cut biopsy needle, liver biopsy was taken under telescopic vision (Figure 4). In cases of biliary atresia, laparotomy was performed to proceed with Kasai portoenterostomy.

## 3. Results

From December 2007 to December 2014, 61 infants with prolonged jaundice were referred to us from the Department of Pediatrics. Thirty-seven males and 24 females were all referred with pale stool and severe skin icterus. The mean age at first admission to pediatric department was 17.0 ± 12.5 (1–42, median 18 days) days and the mean age at referral to Pediatric Surgery Department was 76.3 ± 25.2 (33–157, median 72 days) days. The time period from the beginning of prolonged-jaundice work-up with nonsurgical investigations till surgical evaluation was 59.3 ± 26.4 days (17–141, median 57 days) for all the patients and 62.7 ± 28.3 (21–141, median 56.5 days) days for the patients who had biliary atresia.

The cholangiography technique was laparoscopy-assisted in 23 patients and was via laparotomy in the remaining 38. The technique to be applied was determined by preference of the senior surgeon. When the fibrotic gallbladder was observed, either with laparoscopy (*n*: 5) or with laparotomy (*n*: 9), portoenterostomy was performed without cholangiography. Thirty of 61 (49.2%) prolonged-jaundice patients, who were detected to have biliary atresia, underwent Kasai portoenterostomy. Thirty-one of the patients had patency of ducts. Single-port laparoscopy-assisted percutaneous cholangiography was performed in 18 patients. Six of them were diagnosed to have biliary atresia. The remaining 12 were detected to have patent biliary ducts and tru-cut liver biopsy was performed (Group I). In two infants, an additional 5 mm trocar was needed to be inserted, in order to retract the dilated intestinal loops in one and to occlude the distal choledochus with grasper in an effort to direct the contrast agent flow into the intrahepatic ducts in the other.

**Figure 5:**
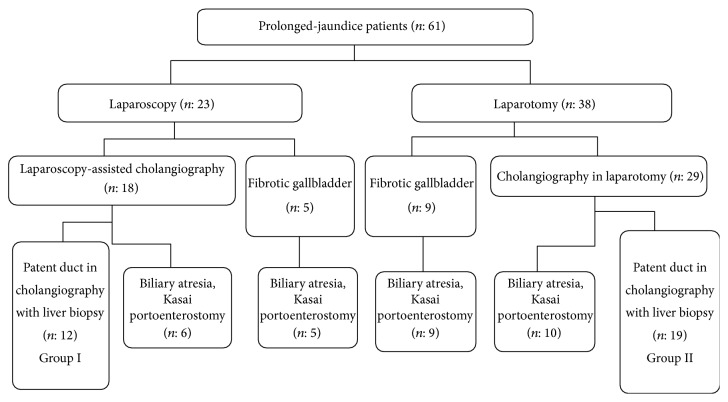
Data flow chart of the referred prolonged-jaundice patients.

Twenty-nine patients had cholangiography via laparotomy; biliary atresia was detected in 10 patients and the operation proceeded to portoenterostomy. Patency of the ducts was observed in 19 patients and they were followed on with liver biopsy (Group II).  Figure 5 shows the data flow chart.

In Group I, 12 (66.7%) of 18 patients had patent biliary ducts confirmed by laparoscopy-assisted cholangiography and unnecessary laparotomy was avoided. In Group II, 19 of 29 (65.5%) patients had patency of the ducts confirmed by open cholangiography.

Table 1 shows the differences between the two groups. The duration of operation was 64.6 ± 32.6 minutes (30–120, median 55) in Group I and 148.4 ± 65.2 minutes (60–300, median 150) in Group II. The difference between the two groups was statistically significant (*p* < 0.001). The postoperative initial feeding time was 44.8 ± 41.8 hours (6–120, median 24 hours) in Group I and 91.0 ± 29.5 (48–168, median 96 hours) hours in Group II. The difference between the two groups was statistically significant (*p* = 0.003). The postoperative full feeding achievement time was 66.3 ± 39.7 (24–144, median 48 hours) hours in Group I and 119.0 ± 30.5 (78–192, median 116 hours) hours in Group II. The difference between the two groups was statistically significant (*p* = 0.001). Postoperative problem-free full feeding achievement parameter presumed the time period of follow-up by pediatric surgeon. In other words, after the achievement of full feeding, the surgical follow-up in pediatric units ended.

## 4. Discussion

The age of the patient with biliary atresia at surgery is the most important determining factor of the postoperative long-term survival. Performing portoenterostomy before 30, 31 to 60, 61 to 90, 91 to 120, and after 120 days has 5-year-survival rates of 62.5%, 43.6%, 39.5%, 28.6%, and 28.8%, respectively [1]. In our series, the mean age at first admission to the pediatrician is 17.0 ± 12.5 (1–42, median 18) days and the mean age during the surgical exploration is 76.3 ± 25.2 (33–157, median 72) days. The mean delay time during pediatricians' nonsurgical investigations is 59.3 ± 26.4 (17–141, median 57) days. Acting by right reason of the invasiveness and morbidity of the laparotomy, pediatricians usually prefer to refer the patient to the pediatric surgeon to exclude the biliary atresia after all their nonsurgical investigations are complete. According to our series, 30 of 61 (49.2%) of prolonged-jaundiced patients had biliary atresia and the remaining 31 (50.8%) had unnecessary explorations, which supports pediatricians' hesitation. On the other hand, for the 30 patients with biliary atresia, there had been a mean delay of 62.7 ± 28.3 days, which can also be considered as wasted time, because avoidance of such a delay and early operation could have meant a longer life expectancy of higher quality.

In 1980, Hirsig and Rickham performed laparoscopy in 9 children with direct hyperbilirubinemia and reported their opinion that cholangiography with liver biopsy can be carried out by laparoscopy within the first 4 weeks of life and pointed out the importance of the early cholangiographic evaluation [2]. In 2000, Hay et al. reported 12 patients with performed laparoscopy-guided percutaneous cholangiogram with single umbilical port and if the cholangiogram excludes the biliary atresia additional 3 trocars were inserted and liver biopsy was taken with intracorporal laparoscopic procedure with a short duration of operative time [3]. In our series, tru-cut biopsy supplied so satisfactory information that we did not need to take liver biopsy intracorporally via inserting additional trocars. In other series, during laparoscopic evaluation, cholangiogram was reported to be performed either by exteriorizing the gallbladder through the incision of the working port [4, 5] or by hitching the gallbladder to the lateral aspect of the abdominal wall [6]. Şenyüz et al. reported the importance of early diagnosis of surgical jaundice in a neonate for surgical success. They performed diagnostic laparoscopy in 24 prolonged-jaundice infants and 10 (42%) of them proved to be neonatal hepatitis that unnecessary laparotomy was avoided [7]. Jancelewicz et al. reported a screening algorithm for the efficient exclusion of biliary atresia in infants with cholestatic jaundice [8]. They proposed a screening algorithm after all standard work-up is done and resulted in being nondiagnostic. This standard work-up included ultrasound, laboratory tests (TORCH work-up, cultures, etc.),  and screening for cystic fibrosis, hypothyroidism, galactosemia, and other metabolic and genetic conditions. We think that all these investigations take a long time that can also be considered as a delayed time.

The weakness of the current study is that we could not compare the groups with the length of stay and cost. We think that the reason is the heterogeneity of the variables from individual patient's comorbidities, referral and discharge practices outside of our clinic within the hospital, precludes us from such comparison.

Our experience suggests that, with shorter duration of operation, more quickly achieved full enteral feeding, and better cosmesis, the pediatricians could confidently refer the prolonged-jaundice patients to the surgery team earlier, without waiting for the results, but during their investigation for the metabolic disorders or infectious disease, so that a timely laparoscopic evaluation could supply an efficient contribution to the patients.

## 5. Conclusion

Etiological investigation of prolonged jaundice in infants is often a hard, time-consuming work for pediatricians. Prognosis in biliary atresia highly depends on the patient's age during operation. Waiting for the results of the investigation for the nonsurgical causes of prolonged jaundice is an important reason of delay of biliary atresia treatment. To prevent such a loss of time, pediatricians can be convinced with early referral of the children with prolonged jaundice to the surgeons.

## Figures and Tables

**Table 1:** Comparison of the groups who were detected to have patent ducts via either laparoscopy (Group I) or laparotomy (Group II). Tru-cut biopsy gave satisfactory information in all patients in Group I. In both groups, no complications were observed either in peroperative or in postoperative time.

	Single-port laparoscopy-assisted cholangiography and liver biopsy Group I	Laparotomy with cholangiography and liver biopsy Group II	*p*
Mean duration of operative time (minutes) (mean ± SD, min-max, and median)	64.6 ± 32.6 minutes (30–120, median 55)	148.4 ± 65.2 (60–300, median 150)	<0.001
Mean postoperative initial feeding time (hours) (mean ± SD, min-max, and median)	44.8 ± 41.8 (6–120, median 24)	91.0 ± 29.5 (48–168, median 96)	0.003
Mean postoperative full enteral feeding achievement time (hours) (mean ± SD, min-max, and median)	66.3 ± 39.7 (24–144, median 48)	119.0 ± 30.5 (78–192, median 116)	0.001
